# Barriers and Facilitators to Implementing Web-Based Dementia Caregiver Education From the Clinician’s Perspective: Qualitative Study

**DOI:** 10.2196/21264

**Published:** 2020-10-02

**Authors:** Anthony J Levinson, Stephanie Ayers, Lianna Butler, Alexandra Papaioannou, Sharon Marr, Richard Sztramko

**Affiliations:** 1 Division of e-Learning Innovation McMaster University Hamilton, ON Canada; 2 Department of Geriatric Medicine McMaster University Hamilton, ON Canada

**Keywords:** dementia, caregiver, online education, implementation science, internet, eHealth

## Abstract

**Background:**

Internet-based dementia caregiver interventions have been shown to be effective for a range of caregiver outcomes; however, little is known about how to best implement them. We developed iGeriCare, an evidence-based, multimedia, web-based educational resource for family caregivers of people living with dementia.

**Objective:**

This study aims to obtain feedback and opinions from experts and clinicians involved in dementia care and caregiver education about 1 iGeriCare and 2 barriers and facilitators to implementing a web-based caregiver program.

**Methods:**

We carried out semistructured interviews with individuals who had a role in dementia care and/or caregiver education in several key stakeholder settings in Southern Ontario, Canada. We queried participants’ perceptions of iGeriCare, caregiver education, the implementation process, and their experience with facilitators and barriers. Transcripts were coded and analyzed using a grounded theory approach. The themes that emerged were organized using the Consolidated Framework for Implementation Research.

**Results:**

A total of 12 participants from a range of disciplines described their perceptions of iGeriCare and identified barriers and facilitators to the implementation of the intervention. The intervention was generally perceived as a high-quality resource for caregiver education and support, with many stakeholders highlighting the relative advantage of a web-based format. The intervention was seen to meet dementia caregiver needs, partially because of its flexibility, accessibility, and compatibility within existing clinical workflows. In addition, the intervention helps to overcome time constraints for both caregivers and clinicians.

**Conclusions:**

Study findings indicate a generally positive response to the use of internet-based interventions for dementia caregiver education. Results suggest that iGeriCare may be a useful clinical resource to complement traditional face-to-face and print material–based caregiver education. More comprehensive studies are required to identify the effectiveness and longevity of web-based caregiver education interventions and to better understand barriers and facilitators with regard to the implementation of technology-enhanced caregiver educational interventions in various health care settings.

## Introduction

### Background

The prevalence of dementia is increasing, and more family caregivers are involved in caring for people living with dementia. Despite their key role, many caregivers may have little knowledge of the disorder, community resources, or the caregiving role. As a result of the impact of dementia on caregivers, the Canadian National Dementia Strategy, Ontario Dementia Strategy, Health Quality Ontario Quality Standards for Dementia, and other clinical guidelines highlight dementia caregiver education as an important component of quality care [1-6]. Most caregiver education is provided face-to-face during a clinical visit. Providing caregiver education in a clinical setting can be extremely challenging because of time constraints. The most common forms of caregiver education include referrals to community organizations (such as the Alzheimer Society), commercially available materials, or customized clinical handouts and pamphlets. Caregivers in rural communities may have no access to dementia specialists, and therefore, no opportunities for face-to-face education.

Internet-based caregiver intervention has emerged as a potential solution to address some of these challenges. A recent needs assessment outlined that caregivers were actively seeking trustworthy sources of information about dementia on the internet [7]. Various systematic reviews suggest that web-based interventions may result in a range of improved health outcomes for caregivers, including reductions in depression, stress, distress, and anxiety [8-11]. Other studies have identified that greater public education is needed for caregivers, and improved mechanisms are needed for busy clinicians to provide caregiver education [12].

We developed iGeriCare (Division of e-Learning Innovation, McMaster University), a multimodal e-learning intervention, to help educate family caregivers of people with dementia. It was developed by experts in dementia and web-based learning as well as family caregivers to help meet the needs of caregivers by improving their knowledge and skills as well as by raising awareness of strategies and services to improve their quality of life and that of the person with dementia. iGeriCare consists of 10 multimedia e-learning lessons, curated resources, a series of weekly *microlearning* emails with small segments of content to reinforce material from the lessons and monthly web-streamed live events that allow participants to post questions to subject matter experts. iGeriCare has been designed to assist health care providers in providing high-quality education efficiently and effectively to caregivers of people living with dementia. We applied best practices in e-learning instructional design, such as the use of instructional graphics, audio narration, and personalization, which have been shown to be more effective than e-learning methods that do not conform to best-evidence instructional design [13,14].

### Objectives

Although web-based education may be an effective intervention, little is known about how best to implement it in various family caregiver education settings [15]. In this study, we performed a qualitative examination to identify recurrent themes, including facilitators and barriers, that might inform other organizations’ planning and implementation efforts with regard to web-based dementia caregiver education. We report on factors affecting the implementation of caregiver education from the perspective of those involved in the clinical care of people with dementia and caregiver education.

## Methods

### Study Design

We conducted a qualitative study consisting of semistructured interviews with 12 individuals involved in dementia care and caregiver education and used a grounded theory approach [16-19]. We chose to use semistructured interviews as opposed to close-ended survey questions to allow participants the freedom to express their views in their own terms. We used the Consolidated Framework for Implementation Research (CFIR) to evaluate factors that could influence implementation effectiveness. It provides a pragmatic structure for approaching real-world issues and themes by bringing together key constructs from published implementation theories [20].

### Setting and Timing

The study was conducted in several key stakeholder health care settings in Southern Ontario, Canada, including family medicine clinics, geriatrics and/or dementia clinics, geriatric psychiatry, and others. The interviews were conducted from October 31, 2018, to March 25, 2019.

### Participants

A total of 12 participants were interviewed, each with a key role in dementia care and/or caregiver education in their organization. Participants provided written informed consent, and the protocol was approved by the Hamilton Integrated Research Ethics Board at McMaster University.

We targeted opinion leaders who actively work with caregivers from a range of disciplines, including geriatrics, neurology, psychiatry, family medicine, and community care. We targeted a wide range of practice settings, including hospitals, outpatient clinics, and advocacy organizations. An internet search was conducted to identify potential participants from a range of disciplines. For convenience, we stayed within Southern Ontario as we wanted to conduct the interviews in person. None of the participants were involved in the development of iGeriCare.

### Data Collection

The interviews took place in or near the participants’ own offices and were conducted by 2 female research team members: a research assistant (SA) and a research coordinator (LB). Both interviewers had extensive experience in conducting interviews. The principal investigator (AL) participated in 2 interviews. Participants were asked to review the iGeriCare website before their interview. If they were unable to review the website before their interview, they were given the opportunity to review it before beginning the interview. The interviewers used semistructured interview questions and asked clarifying questions as needed (Multimedia Appendix 1). A practice interview was conducted during the design of the interview guide. Participants’ perceptions of iGeriCare and collateral implementation tools were explored in particular and web-based dementia caregiver interventions and approaches to caregiver education in general. The interviewers debriefed with the broader research team after each interview to identify the emerging themes and potential areas of exploration and focus for subsequent interviews. The interviews were between 30 min and 45 min in length and were audiotaped and transcribed verbatim. Only 2 research staff members (SA and LB) had access to the file linking transcripts with participants’ identities.

### Data Analysis

Transcripts were analyzed using a grounded theory approach [16-19]. The members of the research team reviewed an initial transcript to generate a list of concepts and domains to determine a preliminary inductive coding scheme [21]. To test the preliminary inductive coding scheme, the research team applied codes to an initial transcript and revised the codes, themes, and subthemes as necessary to yield a final coding scheme by consensus (Multimedia Appendix 2). Overall, 2 research team members then independently reviewed the transcripts and applied the codes to each transcript by labeling phrases on the hard copies. Coding differences between the primary coders were resolved by weekly discussions with the members of the larger research team until a consensus was reached. The coded transcripts were entered into the QSR International NVivo 12 qualitative data analysis software to facilitate coding and analysis of transcripts. The research team members compared the codes within and across interviews to align and map them with the domains and constructs in the CFIR. CFIR has been applied to a variety of other contexts (eg, health care delivery and process redesign, quality improvement, health promotion, and disease management) and health outcomes (eg, mental health, obesity, and blood pressure). It is most commonly applied to gain an in-depth understanding of participant experiences (eg, implementation processes, barriers, and facilitators to implementation) in innovation implementation [22].

## Results

### Participant Characteristics

A total of 14 individuals were initially invited to participate; 1 was unavailable for the interview and 1 did not respond to correspondence (Table 1). Of the 12 individuals that participated, 8 provided a substantial review of the iGeriCare intervention before being interviewed, whereas 4 individuals provided little to no review. The participants had the following disciplines or specialty roles: family medicine (n=3), geriatrics (n=3), nursing (n=2), neurology (n=1), geriatric psychiatry (n=1), general internal medicine (n=1), and social science (n=1). A total of 9 participants were physicians. We tried to engage regional opinion leaders; overall, 9 participants were affiliated with the host institution, McMaster University, whereas 3 participants were from other institutions or organizations. Saturation of themes was seen after 12 interviews.

We present the key findings within each of the 5 CFIR domains and the relevant constructs within each domain.

**Table 1:** Participants’ demographic information.

ID	Gender/ sex	Reviewed website before interview	Workplace						
			Division	Role	Setting	Practice location	Dementia or memory clinic	Organization	McMaster affiliation
001	Male	Substantial review	Family medicine	Physician	Outpatient and/or community	Urban	Yes	Health care organization and university	Yes
002	Male	Substantial review	Family medicine	Physician	Outpatient and/or community	Urban	Yes	Health care organization and university	Yes
003	Male	Minimal or no review	Geriatrics	Physician	Outpatient and/or community	Urban	Yes	Health care organization and university	No
004	Female	Substantial review	Geriatric psychiatry	Physician	Outpatient and/or community and inpatient	Urban	No	Health care organization and university	Yes
005	Female	Substantial review	Nursing	Researcher	Outpatient and/or community	Urban and rural	No	University	Yes
006	Male	Minimal or no review	Neurology	Physician	Outpatient and/or community and inpatient	Urban	No	Health care organization and university	Yes
007	Female	Minimal or no review	Family medicine	Physician	Outpatient and/or community	Urban and rural	Yes	Health care organization and university	Yes
008	Female	Substantial review	Geriatrics	Physician	Outpatient and/or community and inpatient	Urban	Yes	Health care organization and university	Yes
009	Female	Minimal or no review	Geriatrics	Physician	Inpatient	Urban	No	Health care organization and university	No
010	Female	Substantial review	Nursing	Researcher	Outpatient and/or community	Urban and rural	No	University	Yes
011	Female	Substantial review	Social science	Coordinator	Outpatient and/or community and inpatient	Urban and rural	No	Nonprofit organization	No
012	Male	Substantial review	General internal medicine	Physician	Outpatient and/or community and inpatient	Urban	No	Health care organization and university	Yes

### Intervention Characteristics

Intervention characteristics refer to the specific characteristics of iGeriCare [20].

#### Theme 1

Theme 1 is as follows: iGeriCare was generally perceived as a high-quality, trusted intervention for caregiver education, with many participants highlighting the relative advantage of a web-based format.

The *design, quality, and packaging* of iGeriCare was perceived by many participants as being expertly bundled, presented, and assembled, noting that it was a resource that was *trusted* and *valuable* [20]:

I really like this, partly because it’s knowledge that has been vetted, so it’s not the same as googling dementia and you really can’t control what comes up and what doesn’t. So, I like the fact that it’s summarized it’s at a level where it is easily digestible, and it’s not something that is difficult for family members.

Participant 001

It’s a very nice-looking website...from what I’ve seen it’s very comprehensive. I mean like, you’re hitting caregiver wellness, you’re hitting apathy, you’re hitting driving—you know, you’re hitting promotion of brain health. I mean, it seems like, I don’t see any gaps just from a superficial look at it. It looks like its gone through multiple passes and stuff. It looks very polished. It seems to me that a lot of work has gone into it.

Participant 006

*Relative advantage* refers to the participants’ perception of the advantage of implementing iGeriCare versus an alternative solution [20]. Most participants perceived the web-based format and increased ease of access to facilitate dementia education for a wider caregiver audience as a *relative advantage* when compared with traditional current practice or formats:

I think with the videos and that sort of thing [iGeriCare] is a much better alternative. It’s something that allows them to sit and watch and say, ‘oh that’s a digestible portion of information that I can take.

Participant 001

I think it is important. We can’t possibly educate everybody about all of this in the context of clinic nor does it always feel like the right place for it. People just sometimes need to learn on their own at home, and then come back with questions once they’ve had a chance to be exposed to it.

Participant 002

The hundreds of people that I’ve heard say in an education series, ‘I wish my brothers were here’, or ‘I wish my father would have joined me.’ And they’re not coming through our door, and they’re not going to their local chapter, or if they live in another part of the province—that they can access [iGeriCare].

Participant 011

A few participants did, however, voice concerns about the format, noting that much of their current caregiver education was delivered with more traditional approaches such as face-to-face delivery or printed pamphlets. In addition, there remains a perception that older adults do not use the internet or search the web for information:

Many of the older persons that we deal with are either not really that computer-savvy, maybe they spend a little bit of time on the internet and might play some games on their computer, but many of them don’t use it to search for information. I think that’s a younger generation kind of thing.

Participant 007

#### Theme 2

Theme 2 is as follows: iGeriCare is perceived as being readily usable, with minimal disruption to existing workflows, and it can be customized or revised as needed.

The iGeriCare intervention was seen to have minimal barriers to immediate implementation, aligning with the CFIR construct of *trialability*:

It will be helpful...I can see us having it up during our memory clinic.

Participant 002

I am thrilled, this is really phenomenal; I’m going to immediately start using this.

Participant 006

We’re already using it. We have the [iGeriCare educational prescription pad], and I give it to families as I’m talking about supports.

Participant 008

The overall construct of *intervention characteristics* was perceived positively by most participants.

### Outer Setting

The outer setting is the economic, political, and social context within which an organization resides [20]. The outer setting influences implementation and is often affected by changes in the inner setting.

Theme 1 is as follows: iGeriCare was seen to meet patient needs because of its alternative format and because the flexibility of *on-demand* web-based learning helps overcome the barrier of time constraint for both clinicians and caregivers.

The *patient needs and resources* construct identifies the extent to which organizations understand the barriers and facilitators of meeting patient needs as well as their ability to prioritize those needs [20]. Patient needs were identified as an important outer-setting construct that could drive demand for services and facilitate participant support for implementing the intervention:

[Education] is a lot of “here are some pamphlets,” and a lot of relying on the caregiver or on the person who may have a Mild Cognitive Impairment diagnosis to go on and sort of read for themselves. So, it can be a little overwhelming...it’s a lot of text and sometimes you can get overwhelmed...by the end of that hour and a half, both of them are tired right, and so something like this [iGeriCare] is great to say, “Here, you don’t need to try to remember everything I said, I really think you should read this and this, and when I see you again in 6 months, we can answer any questions.”

Participant 001

I definitely think that there’s obviously a need. Some people don’t like to go to a [location] to be with other caregivers, that’s not how they learn.

Participant 008

As noted above, patient and/or caregiver needs were seen to be met through the increased ease of access for a wider audience than traditional education practices currently in place. Health care provider participants highlighted the importance of having alternative resources available for delivery to patients and families.

### Inner Setting

The inner setting refers to the provider’s specific practice setting and includes features of structural, political, and cultural contexts through which the implementation process will proceed [20]. Our participants were selected specifically because they were leaders and decision makers in their health care settings and could provide insight into existing workflows.

Theme 1 is as follows: Most participants saw the iGeriCare intervention as a good fit with their existing workflows. Conversely, a few participants expressed concerns about its implementation within their practice settings and existing workflows.

Many participants stated that iGeriCare was presently being used or could easily be implemented because of its *compatibility* and *relative priority*. *Relative priority* refers to the individual’s perception of the importance of implementation within an organization [20]:

I think that it would definitely streamline my practice. Because I know that it’s one resource that I can trust, and I don’t need to be looking for.

Participant 001

If I have a patient with dementia and I meet with the family I would say, “there’s a nice program [iGeriCare] that you could look at, go look at it and then when you come back to see me, later on, we can go over things that you don’t understand.”

Participant 012

*Implementation climate* identifies the stage of change an organization is in, how receptive individuals are to an intervention, and the extent to which it will be supported in the organization [20]. Within this construct, some participants identified barriers to the implementation of web-based education because of their current practices or concerns regarding caregiver demographics:

I give my overall framework for the patient, I then give them this Alzheimer’s Society pack, with lots of information...and I give them a referral sheet.

Participant 003

I think we do a lot of that already via other ways, and I think that for the right person, I could see perhaps if it were a younger caregiver who was looking for more detailed information, perhaps that might be something we might include—but I don’t think I would.

Participant 007

### Characteristics of Individuals

Characteristics of individuals includes aspects that impact the individuals involved in the intervention and/or implementation process [20].

#### Theme 1

Theme 1 was as follows: Many participants were familiar with the intervention and felt confident of their ability to implement iGeriCare.

*Knowledge and beliefs about the intervention* refers to the participant attitudes toward and value placed on the intervention as well as familiarity with facts, truths, and principles related to it [20]. Our findings showed that individuals who had taken the time to review iGeriCare were more positively predisposed toward it and placed a higher value on the intervention than those who were less familiar with it:

I thought it was high quality, overall very useful.

Participant 004; substantial review of the intervention

It would certainly fit with the National Dementia Strategy.

Participant 010; substantial review of the intervention

I think it’s great that people can go on, listen again to a session that they might have already done, share it with family and friends so there’s consistency in messaging. We want to get everyone within a family network or small community on the same page if you will.

Participant 011; substantial review of the intervention

#### Theme 2

Theme 2 was as follows: The relationship of the participants to the iGeriCare developers’ institution affected their degree of commitment to the intervention.

Participants did not specifically reference *individual identification with the organization*, a broad construct related to how individuals perceived the organization and their degree of commitment to it [20]. Rather, this was expressed as a sense of pride in their organization’s current educational practices. Individuals who were more clearly identified with the organizations of the developers of the intervention were more positively predisposed to the intervention and its implementation. Individuals who did not clearly identify with the organizations of the developers were less predisposed to the intervention and its implementation.

### Process

A successful *implementation process* typically requires an active change process aimed at achieving individual- and organization-level use of the intervention as designed [20].

#### Theme 1

Theme 1 was as follows: Most participants felt that they could implement iGeriCare using collateral promotional materials or by sharing the website’s URL.

Most participants were confident of their ability to implement iGeriCare according to plan; this aligns with the CFIR construct of *executing* [20]. Participants commented on the need to give something to the families to go home with and praised the preexisting iGeriCare promotional materials that were available:

I think the only way that I can easily pass this information on to patients and their families is if I had something in my hand that I could give them to go away with. Whether it’s a card or a link to a website something that can say, “I can vouch for this, this is a good resource, I need you to look at this.”

Participant 002

This is great [iGeriCare educational prescription pad], this is so easy you know it’s something that can be ready to pull out for every patient.

Participant 004

Although most participants felt that they could easily implement iGeriCare, one barrier identified was the need for a constant reminder about the resource and keep it front of mind to the organizations and individuals:

In primary care there are barriers to any new resource or any new community program and the biggest one is just the “noise”—the sheer number and volume of programs and tools and resources that are coming at us.

Participant 002

#### Theme 2

Theme 2 was as follows: Participants suggested several strategies to continue engaging stakeholders, including finding champions, engaging others in the circle of care, presenting at medical conferences, and incorporating the resource into various health professions’ curricula.

Participants commented on the importance of attracting and involving appropriate individuals in the implementation and use of the intervention through a combination strategy of social marketing, education, role modeling, training, and other similar activities, which align with the CFIR construct of *engaging* [20]:

I do think it requires someone that is a champion that can bring it in and talk to the benefits of it. And I think when people kind of see how this can match their learning gaps or their knowledge gaps, then that’s when you are going to get it to pick up for that.

Participant 001

That might be something good to send back to the family doctor to say, “look, I’ve recommended these things for your families and I think that they many actually come to talk about. Just so you know these are the resources,” and to have that, so the family doctors are aware of, “maybe I should take a quick look at what’s gone on,” and things like that. [Participant 001] 

It might help with Alzheimer Society’s or First Links navigators, where a lot of this one-to-one peer education may be saved by helping people go through this, but I think it could certainly augment the care that’s being provided, and it might help provide again support that actually might save some of the [the time of] allied health staff.

Participant 003

What about the family docs, are you going to be explaining it to them? That’s where the patients really are...

Participant 012

In addition to the above-mentioned barriers and facilitators related to the implementation of iGeriCare within existing clinical workflows, we also discovered broader insights into the implementation of web-based education. Participant-identified barriers and facilitators related to the implementation of web-based educational interventions for caregivers related to CFIR constructs are summarized in Textbox 1.

**Participant-identified barriers and facilitators related to implementation of web-based educational interventions for caregivers.**

Intervention characteristics

- Facilitator
  - The design quality of the intervention, in part because of its simplistic layout, large icons, minimal effort
  - The intervention is easily implemented in everyday workflows and allows health care providers to trial with users before committing
- Barrier
  - Skepticism about the relative advantage of the web-based nature of the intervention
  - The intervention source being seen as externally developed

Outer setting

- Both facilitator and barrier
  - The format of the intervention being web-based is variably perceived as both a facilitator and a barrier. There is tension between health care providers as some have a positive opinion of the web-based format and others will not recommend because of concerns that the format might not be useful for some caregivers

- Facilitator
  - The content and format are perceived to be aligned with caregiver needs
  - Some networking with other external organizations (ie, Alzheimer Society, hospitals, memory clinics, family health teams)

- Barrier
  - The lack of language options, cultural adaptations, and alternative formats (ie, print)
  - The lack of external policy and incentives to encourage adoption

Inner setting

- Facilitator
  - The intervention easily fits into and is compatible with existing workflows
  - Some settings have a higher relative priority than others for implementation
  - Access to knowledge and information

- Barrier
  - Health care provider concerns over the amount of time it would take to review materials before recommending to patients and families. Lack of integration with electronic medical records
  - Lack of tension for change
  - Lack of organizational incentives and rewards

Characteristics of individuals

- Both facilitator and barrier
  - Level of knowledge about the intervention

- Facilitator
  - Identification with the developer organization
  - Tech-savviness

- Barrier
  - Identification with an external organization
  - Technophobe and/or assumes older adults do not use the internet

Process

- Facilitator
  - Ease and enthusiasm to execute
  - Existing promotional materials
  - Existing champions and opinion leaders

- Barrier
  - Needs ongoing campaigns to maintain awareness of resource
  - Needs constant reminders
  - Costs of promotional materials
  - Costs of attending conferences and/or identifying and promoting resource to new champions

## Discussion

### Principal Findings

In this study, experts in dementia care provided detailed feedback about iGeriCare as well as on barriers and facilitators to implementing a web-based dementia education program for caregivers in general. iGeriCare aligns with the paradigm of shared decision making and the health care triad (the term *health care triad*, with regard to iGeriCare, refers to the person living with dementia, the informal caregiver, and the clinician educator and/or health professional). It can be a meaningful resource to complement face-to-face or print-based educational methods. Participants who reviewed iGeriCare in more depth and identified more with the organization that developed the intervention were more positive about the intervention and enthusiastic about its adoption and/or implementation. In addition to the design quality and credibility of the intervention, participants felt that a web-based intervention could be easily introduced and integrated into existing clinical workflows. Some participants strongly believed that older caregivers do not use the internet and were generally more skeptical about the value of web-based interventions compared with that of more traditional methods and formats. There were a variety of suggestions regarding the process of implementation and ongoing dissemination.

This study adds to the growing body of literature on web-based interventions for caregivers of people with dementia; in particular, it is one of the few studies to examine implementation. Despite the increase in research in this area—it has been estimated that the number of publications in this field increases by 13% each year, including several systematic reviews—we could find very little research published regarding the implementation of web-based caregiver interventions [9-11,23-26]. Moreover, many of the interventions described in the literature do not appear to be more widely accessible outside of their research context. To our knowledge, no previous research has used the CFIR framework to study web-based caregiver interventions.

### Barriers and Facilitators to Web-Based Caregiver Education

We found several potential barriers and facilitators for the implementation of web-based caregiver education tools in clinical practice.

#### Intervention Characteristics

Participants appreciated the instructional design and high-quality web design of iGeriCare, features that are rarely described in the published literature. Web-based caregiver interventions could be quite heterogeneous and could include different components such as health information, education, peer support, professional support, web-based monitoring, or combinations of these components [11]. In the literature, educational interventions rarely describe their instructional design or report whether they conform to the best practices in multimedia learning. Of the available web-based interventions for caregivers of people with dementia, many focus on peer support, contact with a health or social care provider, decision support, and psychological support [26]. Few interventions focus solely on the provision of education or information to caregivers. Of the interventions that focus solely on the provision of education, most are no longer accessible to the public, which makes it challenging to assess the quality of the web-based educational resource.

We found that participants were more favorably predisposed to the intervention and its implementation if they identified with the organizational developers of iGeriCare. This aligns with the CFIR construct of *intervention source*, for example, the perception of key stakeholders about the source of the intervention—whether the intervention is externally or internally developed [27-29]. For some *external* participants, the intervention adaptability, design quality, and relative advantages outweighed the potential barrier of being an externally developed intervention. In other instances, organizations may be hesitant to recommend an intervention from an external source for a variety of reasons, including lack of trust, technical concerns, cultural factors, peer pressure to develop their own version, or fear of directing users to an external site and losing donations.

#### Outer Setting

Most participants felt that providing caregiver education in a web-based format could reduce the gap for family caregiver support and help meet their needs, consistent with the literature [26]. Caregivers of people with dementia may favor reliable web-based education because of the lack of time for face-to-face education, concerns with privacy and stigma, or challenges with travel and arranging care for their care recipient [25]. Several studies have looked at caregiver experiences with web-based educational resources and reported that caregivers value the convenience and flexibility that web-based education provides [30-34]. The concept of clinicians *prescribing* iGeriCare resonated with the participants. This concept of *educational prescription* may also resonate with caregivers who perceive their health care providers as the foundational source of health information and are more likely to engage with high quality, provider-vetted web-based resources [12,35].

However, there were some more ambivalent opinions about whether the web-based format was optimal to meet the needs of caregivers; in particular, a couple of participants felt that older adult caregivers did not use the internet for health information. Despite encouragement from various provincial, national, and global guidelines and quality standards encouraging and referring to the use of web-based education for older-adult caregivers, it is challenging to change the attitudes of potential intervention agents about educational methods and formats. Internet usage of Canadians aged 65 years and above doubled from 32% to 68% between 2007 and 2016, a trend that is expected to continue given the high rates of internet usage by those aged between 45 and 64 years [35,36]. Recent studies support the fact that family caregivers are avid health information seekers [37]. However, an analysis of US caregiver survey data found that dementia caregivers reported somewhat lower levels of health-related internet usage compared with the general public [38]. Caregiver age, education level, and/or income as well as stress caused by caregiving were all shown to influence internet usage in that study. Raising awareness among clinicians with regard to the older caregivers’ use of the internet may also increase their adoption and/or incorporation of web-based resource provision into their practice.

One finding of interest relates to the fact that none of our participants mentioned any external policies or incentives that might drive decisions about adoption. This is interesting given the recent dementia quality standards that promote caregiver education. More incentives might be an external force to help influence and encourage the implementation of effective web-based caregiver educational interventions [28,39].

#### Inner Setting

Our findings that most participants saw iGeriCare as a good fit with their clinical workflows and were keen to implement the intervention are aligned with the research around the constructs of compatibility of the implementation climate, the relative priority for caregiver education, and readiness for implementation. Participants represented a range of different clinical practice settings and disciplines with different structural characteristics. This did not seem to impact their perceptions of the intervention or desire to implement. Many of the participants were affiliated with the same organization—McMaster University—an organization with a relatively flexible culture that embraces innovation. Culture has been shown to have a significant influence on the implementation effectiveness and may help explain the enthusiasm for the intervention among participants from within this organization [40,41].

Most participants enthusiastically voiced their readiness for implementation. This is consistent with the elements of iGeriCare, such as ease of access to digestible information, knowledge about the intervention and how to incorporate it into work tasks, and the level of resources required to implement the intervention [27,42-44]. Very few resources are required for implementation, and most participants felt that they could implement iGeriCare by using the available collateral promotional materials (eg, poster, educational prescription pads) or just by sharing the website URL with the caregivers. Participants from primary care acknowledged that they were inundated with recommended resources; however, strategies to better integrate resources into workflows were identified as essential. Some participants were also enthusiastic about less reliance on print-based promotional materials and voiced an interest in electronic *educational prescriptions*, as long as the process was at least as efficient as traditional methods.

The amount of time an organization has to spend reviewing and approving a new web-based resource and the current culture of the organization are potential barriers to the implementation of web-based caregiver interventions. However, web-based educational interventions can align with existing workflows and can in turn help overcome barriers such as time constraints. Our finding of readiness to implement may also reflect the fact that our participants were predominantly leaders with decision-making power and/or self-efficacy to implement the intervention. Leadership engagement with the support of clinic administration and physicians is critical for the successful implementation of caregiver education delivered on the web [42,43,45-48].

#### Characteristics of Individuals

Our findings reflected the importance of 2 constructs related to the characteristics of individuals: (1) individual identification with the organization and (2) knowledge and beliefs about the intervention. Individual identification is a broad construct related to how individuals perceive the organization and their relationship and degree of commitment to that organization. These attributes may affect the willingness of staff to fully engage in implementation efforts or use the intervention [49,50]. We found that participants who identified more with the organization that developed the intervention were more enthusiastic about implementation, although some of this may also reflect the construct of *intervention source* (as noted above), where they felt that the intervention was *internally developed*.

The construct of knowledge and beliefs about the intervention was particularly relevant. We found that participants’ knowledge about the intervention itself and opinions about older adults’ usage of web-based health resources were important factors in their perception of the intervention and its implementation. Participants with little familiarity with iGeriCare or those who did not think that older adults used the internet were much less likely to consider the implementation. Individual clinician attitudes about web-based caregiver education may not be based on evidence but rather on personal opinions of preference for the format of delivery.

Many participants were physicians. The characteristics of individuals and their knowledge and beliefs about interventions may be particularly important constructs in contexts where physicians are the primary implementation agents, as they tend to have a lot of autonomy with regard to implementing interventions, especially within certain practice settings (such as ambulatory clinics or more *private practice* type of settings). Many physicians in independent practice might not strongly identify with their affiliated health care organizations (eg, hospitals), highlighting the added importance of individual characteristics as a construct in an organization’s potential implementation that may rely on physicians.

#### Process

With regard to the CFIR domain of *process*, participants spoke predominantly of the constructs of *executing* and *engaging*. The quality of execution relates to several factors, including fidelity of implementation, intensity, timeliness of task completion, and engagement of key stakeholders in the implementation process [51,52]. Our finding that most participants felt that they could implement iGeriCare immediately by using collateral promotional materials that had been developed or simply by sharing the website URL with caregivers suggests that the simplicity of our intervention’s implementation was another major facilitator of adoption. Very little additional planning was needed to implement the intervention. This lack of complexity as a facilitator of implementation is consistent with recommendations of other health technology implementation frameworks [53-55].

One of the most challenging elements of process relates to the construct of *engaging*, “attracting and involving appropriate individuals in the implementation and use of the intervention through a combined strategy of social marketing, education, role modeling, training, and other similar activities” [28]. Implementation of a resource is heavily dependent on the enthusiasm of users and adopters. It is almost never a *one-and-done* process but is more of an ongoing campaign that requires constant reminders to existing champions and opinion leaders. This is particularly the case with web-based caregiver education that is not organizationally mandated. Participants had many suggestions regarding how to continue to engage health professionals; these were mostly through continuing professional development conferences or integration into health professional learners’ curricula. Most likely, a multimodal engagement strategy is required, targeting organizations, clinicians, trainees, and family caregivers. The costs associated with ongoing promotion and engagement (whether they be marketing costs, personnel, or the true costs of time for champions) are not trivial and may prove to be an important barrier with regard to the implementation of web-based caregiver education that is not embedded within some type of centralized strategy.

### Limitations

Some limitations should be considered when interpreting the results. First, the recruitment of professionals to the project was limited to those residing in Southern Ontario, which might have led to an underrepresentation of key stakeholders in the discussion. Second, it might also be a limitation that several stakeholders were directly affiliated with the same organization as the developers of the intervention. However, local and regional implementation of iGeriCare was a key goal of the project; therefore, understanding the attitudes of local opinion leaders was important. We also tried to recruit from a range of different disciplines. Challenges for coding consensus have been identified as a limitation of the CFIR because of the large overlap of constructs within and between domains [56]. Another limitation in the application of the CFIR model to web-based interventions identified is the unidirectional (traditional face-to-face) process of implementation [56]. The implementation of iGeriCare needs to be investigated longitudinally to analyze its long-term effects on organizations, professional roles, ways of working, and ultimately on caregiver and patient-related outcomes.

### Conclusions

In summary, we found that opinion leaders in dementia care were generally enthusiastic about implementing high-quality web-based dementia caregiver education. Key facilitators included the quality of the design of the intervention, ease of implementation, and value added for both the health care system and caregivers. Key barriers included the perception that the intervention came from an external source or organization; lack of policy incentives; current normative professional behaviors around health teaching and/or caregiver education; individuals’ knowledge of the intervention and opinions about older caregivers’ usage of the internet; and the costs and challenges with regard to ongoing engagement, awareness raising, and promotion of the intervention. Despite an increase in the number of interventions and research on web-based caregiver interventions, there is very little work to date describing their implementation. Frameworks such as CFIR and others are helpful in delineating the various domains related to implementation of web-based caregiver interventions. Further research with regard to the specific implementation of caregiver education interventions would be beneficial, given the increasing development of these interventions.

Our results have led us to increase the dissemination of collateral promotional materials, continue engagement with various champions and intervention agents, and continue ongoing multimodal strategies for implementation. A new educational prescription web application for clinicians is being field-tested. This innovation may help determine the reach of the intervention, in addition to providing other measures of whether the educational prescription gets *filled* by the caregiver as well as some data related to the *dose* of the educational intervention.

## Appendix

Multimedia Appendix 1 Qualitative interview guide.

Multimedia Appendix 2 NVivo coding tree export.

## Abbreviations

CFIR: Consolidated Framework for Implementation Research
