# Supplementary material for: Barriers and Facilitators to Implementing Web-Based Dementia Caregiver Education From the Clinician’s Perspective: Qualitative Study
Source: JMIR Aging. 2020 Oct 2;3(2):e21264. doi: 10.2196/21264 (PMC7568210; doi:10.2196/21264)
Supplement: Multimedia Appendix 1 [file aging_v3i2e21264_app1.docx]

**INTERVIEW GUIDE FOR HEALTH CARE PROVIDERS OR CARE ORGANIZATIONS**

**To be used as a template when performing interviews**

**Demographic Information**

Interview participant initials

Interviewee discipline/area of clinical work/organization

Interview date

Interviewer

The interview is voluntary and strictly confidential.

The interview will be audio recorded for future content analysis. Your name will not appear on the interview.

**Initial Open Ended Questions**

1. Now that you’ve had a chance to review the iGeriCare website, what did you think of it?
2. How would you use it in your clinical work/organization?
3. How would you promote it to patients/clients or your patients’/clients’ caregivers?
4. Do you see any barriers to using iGeriCare in your practice/organization?
5. What would facilitate using iGeriCare in your practice/organization?

**Follow up questions related to facilitators:**

**Dissemination/Promotional Materials**

What types of brochures or other promotional materials would be helpful for you?

- Printed brochures
- Printed posters
- Educational prescription pad with URL (tearaway)
- PDF’s of resource information that could be printed on demand (black & white vs. colour)
- Other online ‘self-service’ promotional materials (print on demand; re-order on demand)
- Waiting room tv promo video

**Educational Prescription App**

We’ve wondered about an ‘Educational Prescription app’ which administrators, clinic staff, or clinicians might use to email the link to iGeriCare to a caregiver.

- How do you currently recommend web-based resources to patients?
- Do you ever email them a link?
- Would you consider using some type of ‘Educational Prescription app’ to ‘prescribe’ iGeriCare?

**Professional Development**

Would you be interested in accredited/certified e-learning about iGeriCare? Do you think this would help your colleagues to learn about it?

**HQO Standards: Individualized Care Plans and Education/Training**

Health Quality Ontario has recently recommended that all patients with dementia receive an Individualized Care Plan to help guide their care, as a key standard for high quality dementia care. The plan identifies their individual needs, those of their caregivers, and goals of care. As another quality indicator/standard, they state that people living with dementia and their caregivers should have access to education and training on dementia and available support services.

- Do you currently use Individualized Care Plans in your practice?
- How do you currently provide education and training on dementia and available support services to caregivers in your practice? How do you document this?
- Would you consider including iGeriCare as part of an individualized care plan to achieve caregiver education and training goals?

**Project KPI-related Questions**

As part of our project, we have a series of ‘key performance indicators’ in terms of impact of iGeriCare. We’d like to ask you a few questions related to these now.

*(Use future tense if clinician/organization has not yet started using iGeriCare.)*

**Improved confidence in care**

Would you say that 'prescribing' iGeriCare for caregivers in your practice has increased (or will increase) your confidence that you are providing better caregiver education and training?

**Improved client experience**

Would you say that ‘prescribing’ iGeriCare offers (or will offer) caregivers in your practice an improved experience *(e.g., treated with more respect, listened to more, received more care and attention)*?

**Improved Quality of Life**

Do you think iGeriCare has improved (or will improve) caregiver or patient/client Quality of Life?

**Cost effectiveness**

Do you feel that ‘prescribing’ iGeriCare saved (or will save) you time?

How much time - or other resources (e.g. other staff time) might you normally spend on caregiver education?

Do you think using iGeriCare resulted in a more effective use of your time?

**Closing Questions**

Do you have any suggestions to improve iGeriCare?

- Do you feel there are any key content gaps that should be addressed with lessons or resources or live events or the online discussions?

Anything else that we haven’t discussed with respect to facilitating use of iGeriCare in your practice or organization?

Thank you very much for your time today. If you would like, we can alert you when the results of this research are complete, so that we can share the research results with you. We can also alert you when new features or resources are available, such as the online accredited CPD course.

If you have any other questions about the study, please do not hesitate to contact either the research coordinator or local principal investigator.
