# Supplementary figures and images for: Barriers and Facilitators to Implementing Web-Based Dementia Caregiver Education From the Clinician’s Perspective: Qualitative Study
Source: JMIR Aging. 2020 Oct 2;3(2):e21264. doi: 10.2196/21264 (PMC7568210; doi:10.2196/21264)

NVivo coding tree export.


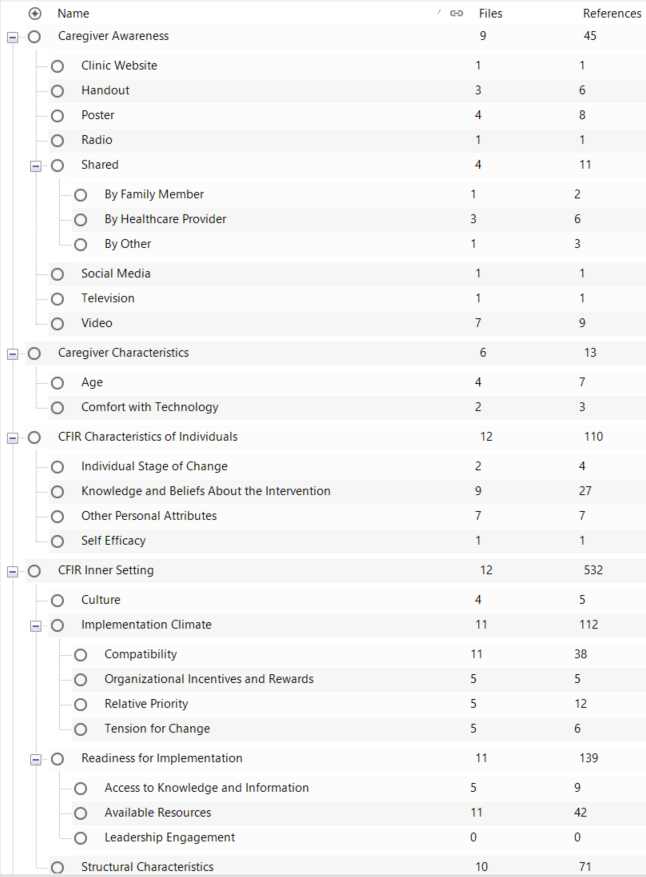


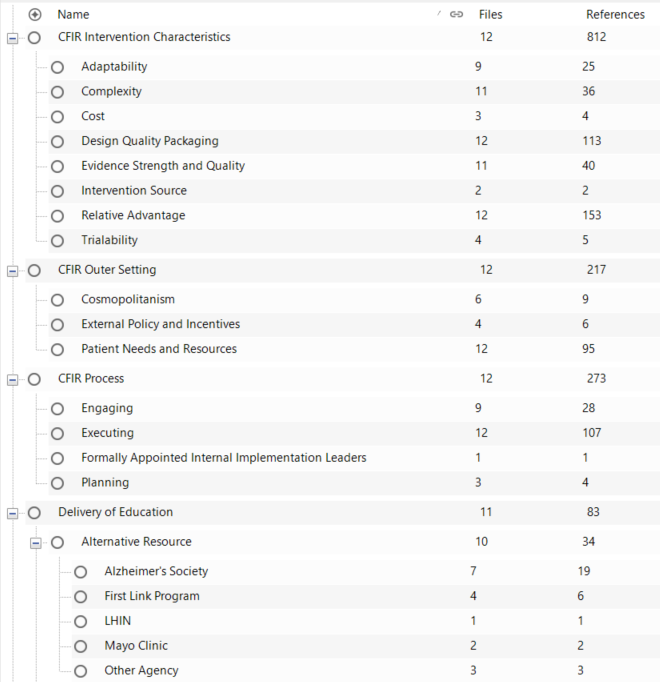


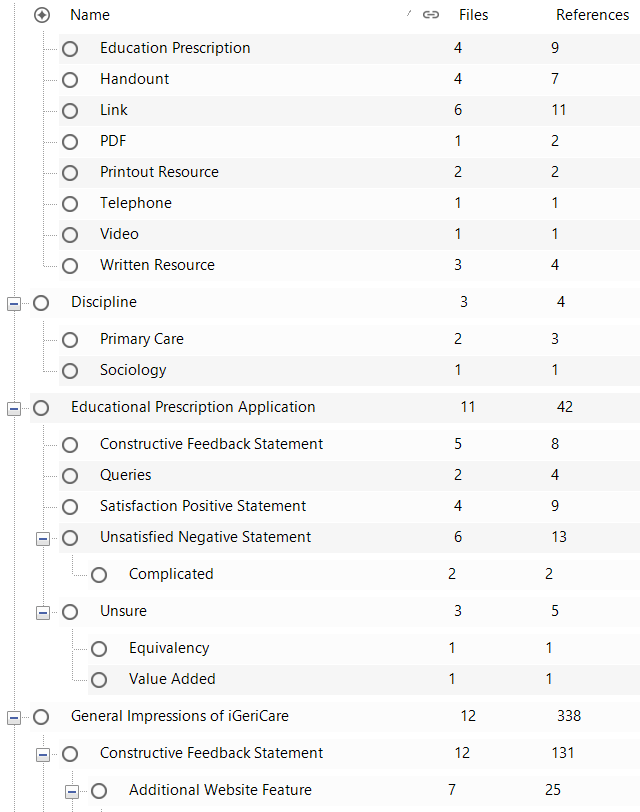


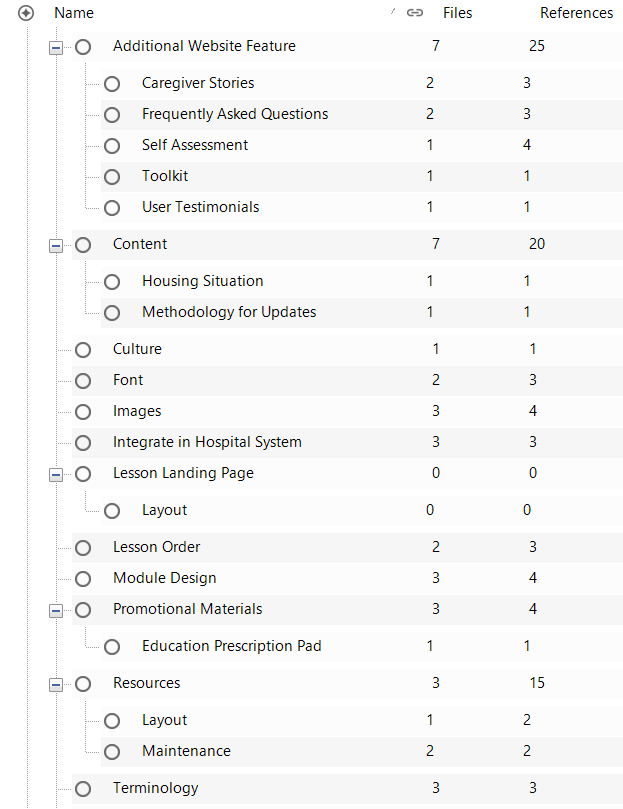


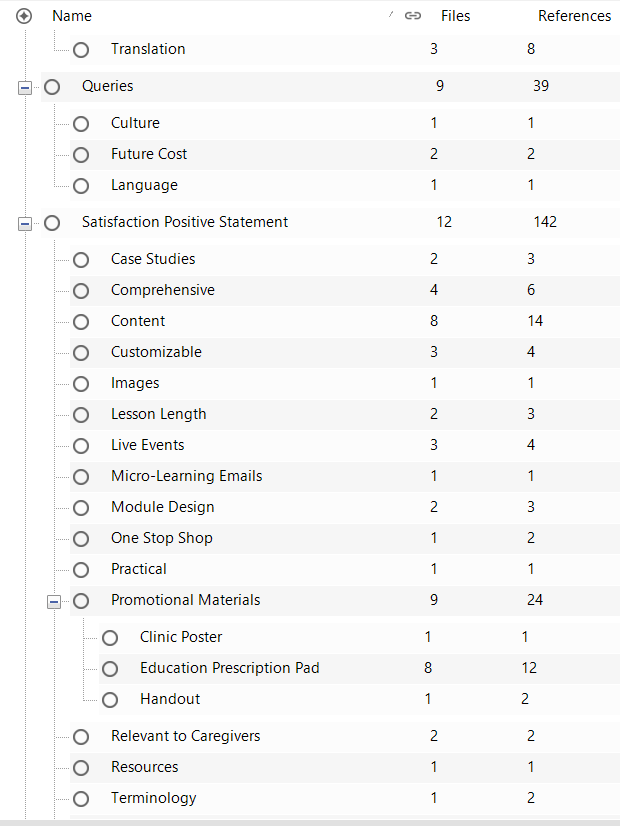


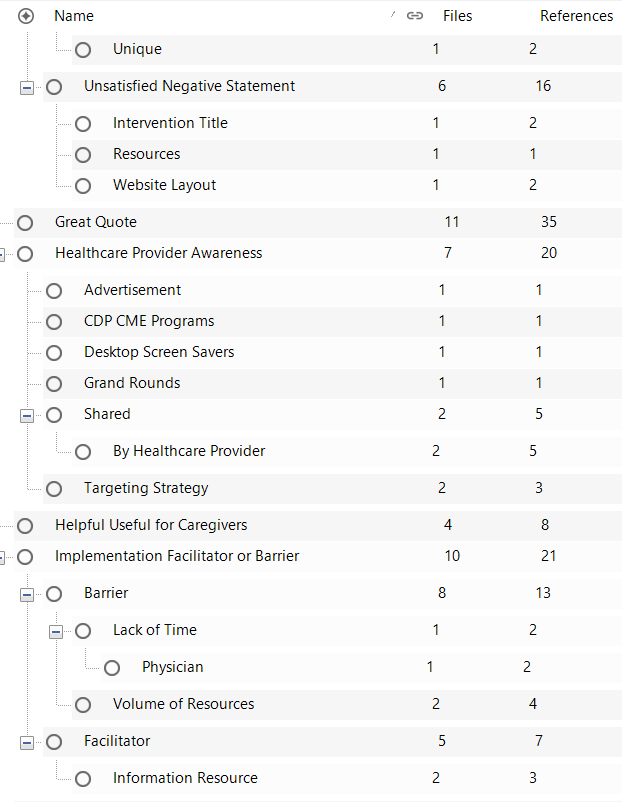


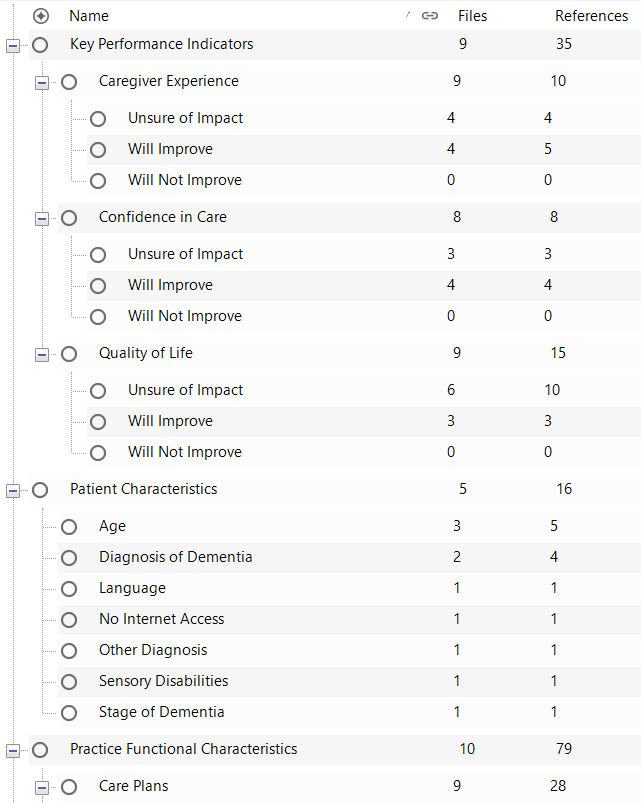


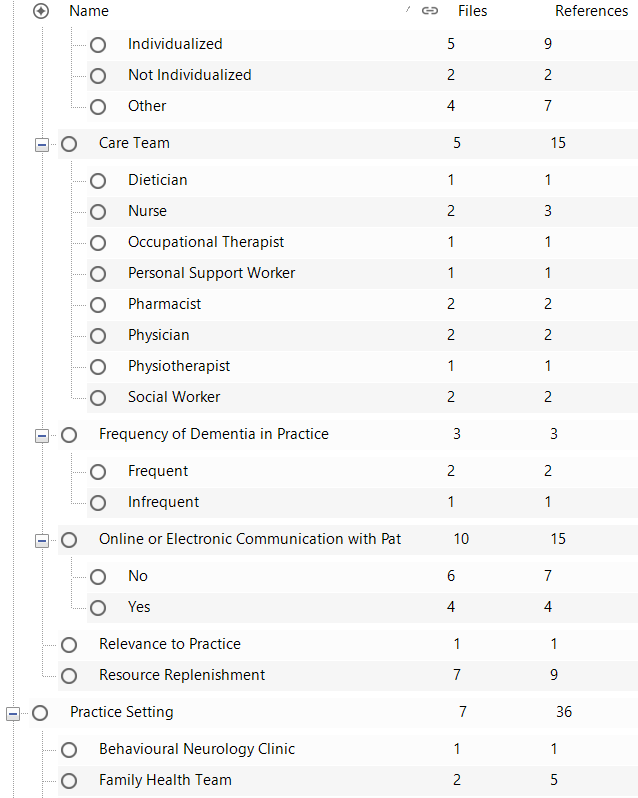


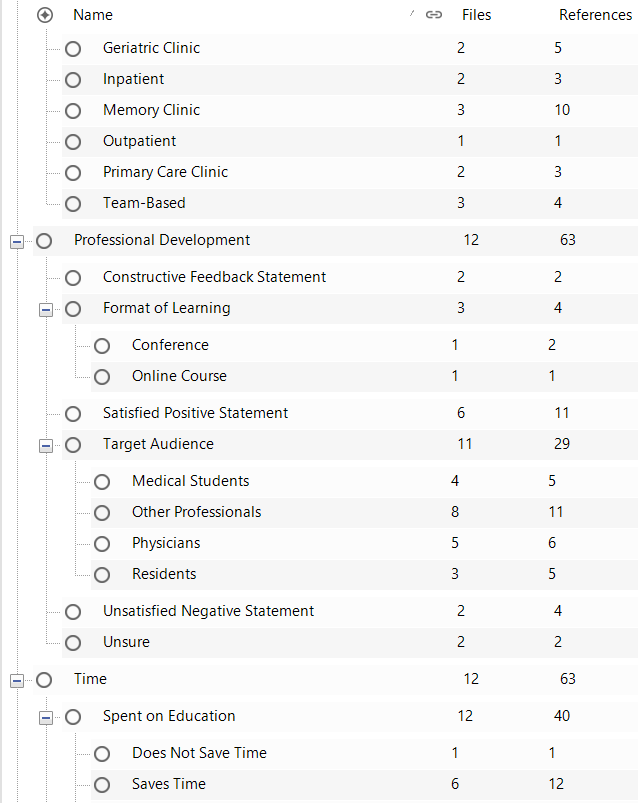


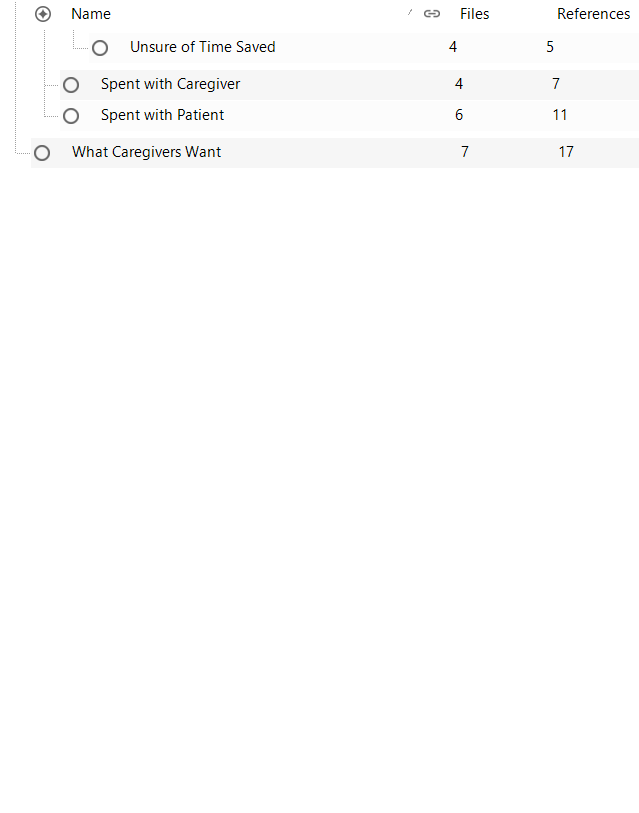

Supplement: Multimedia Appendix 2 [file aging_v3i2e21264_app2.docx]
